# The Aurora Kinase Inhibitor TAK901 Inhibits Glioblastoma Growth by Blocking SREBP1-Mediated Lipid Metabolism

**DOI:** 10.3390/cancers14235805

**Published:** 2022-11-25

**Authors:** Xiudan Zhan, Ru Qiu, Yi He, Zijin Zhao, Meng Huang, Qing Liu, Feng Zhi, Wenyong Long

**Affiliations:** 1 Key Laboratory of Stem Cells and Tissue Engineering (Ministry of Education), Zhongshan School of Medicine, Sun Yat-sen University, Guangzhou 510275, China; 2 Department of Neurosurgery, Xiangya Hospital, Central South University, Changsha 410008, China; 3 Department of Neurosurgery, The First People’s Hospital of Changzhou, Changzhou 213000, China

**Keywords:** TAK901, glioblastoma, lipid metabolism, SREBP1, glioma stem cells, metabolic alterations

## Abstract

**Simple Summary:**

Glioblastoma (GBM) is the most common and aggressive malignant primary brain tumor. However, the therapeutic efficacy for GBM remains unsatisfactory. In this study, we found that TAK901, an Aurora kinase inhibitor, reduced the cell viability of both the GBM cell line and glioma stem cells (GSCs), caused cell apoptosis and considerably inhibited GBM growth in vivo. TAK901 downregulated fatty acid metabolism and cholesterol homeostasis pathways, which played an important role in GBM. Sterol regulatory element-binding protein 1 (SREBP1) overexpression alleviated the TAK901-mediated suppression of cell viability and apoptosis in GBM cells. Taken together, TAK901 is a promising therapeutic approach for GBM.

**Abstract:**

Glioblastoma (GBM) is the most common and lethal malignant primary brain tumor. The standard treatment for GBM including surgical resection followed by radiation therapy and adjuvant chemotherapy with temozolomide remains unsatisfactory. In this study, we investigated the effects of the Aurora kinase inhibitor, TAK901, in GBM both in vitro and in vivo, and explored its key downstream targets. The effects of TAK901 were investigated using cell viability, cell apoptosis, live/dead, cell cycle, Transwell, 3D cell invasion, neuro-sphere, and self-renewal assays. Mechanistic studies were conducted using RNA-seq, lipid measurements, reverse transcription-quantitative polymerase chain reaction (RT-qPCR), and Western blotting. The in vivo efficacy of TAK901 was validated using orthotopic xenograft GBM mouse models. In both GBM cells and GSCs, TAK901 remarkably reduced cell viability, self-renewal, migration and invasion and induced apoptosis and cell cycle arrest. Treatment with TAK901 considerably inhibited GBM growth in vivo. RNA-seq and RT-qPCR analyses showed that TAK901 downregulated the expression and activation of SREBP1. Moreover, SREBP1 overexpression alleviated the TAK901-mediated suppression of cell viability and apoptosis in GBM cells. Our results provide evidence that TAK901 inhibits GBM growth by suppressing SREBP1-mediated lipid metabolism.

## 1. Introduction

Glioblastoma (GBM) is the most common and lethal malignant primary brain tumor, with a poor 5-year survival rate of approximately 7.2% [1,2,3]. The standard treatment consists of surgical resection followed by radiation therapy and adjuvant chemotherapy with temozolomide [4,5,6]. Despite decades of research, the therapeutic efficacy for GBM remains unsatisfactory. Glioma stem cells (GSCs), also known as glioblastoma tumor-initiating cells (TICs), are self-renewing and highly invasive subpopulations of GBM. GSCs are critical for GBM tumor development, invasiveness, and recurrence [7,8]. A new therapeutic approach to inhibit tumor development and subsequently improve outcome for patients with GBM is in urgent need of development.

Aurora kinases are key mitotic cell-cycle regulators, ensuring the orderly and accurate execution of mitosis and cell division [9,10,11]. Aurora A (encoded by AURKA) localizes to centrosomes and spindle poles and is required for mitotic spindle assembly and centrosome maturation [12]. Aurora B (encoded by AURKB), a chromosomal passenger protein, is essential for the phosphorylation of histone H3, chromosome segregation, and cytokinesis [13,14]. Previous studies have indicated that Aurora A and B kinases are overexpressed in various human cancers and are closely associated with poor prognosis, tumor progression, and genetic instability [10,12,15]. TAK901 is a potent multi-targeted Aurora kinase inhibitor that can induce polyploidy and suppress histone H3 phosphorylation in a dose-dependent manner [16], making it a potential novel therapeutic approach for tumors such as GBM.

Cancer cells alter metabolic processes to sustain their characteristic rapid proliferation. Therefore, high-energy demands and aberrant reprogrammed metabolism are hallmarks of cancer cells [17]. A representative metabolic feature of cancer cells is the Warburg effect, in which tumor cells favor glycolysis over the oxidative phosphorylation pathway even under sufficient oxygen supply [18]. In addition, cancer cells have enhanced lipogenesis, which is tightly correlated with glucose metabolism alterations [19,20]. GBM cells tend to utilize enhanced de novo lipid biosynthesis and exogenous lipid intake to fuel ferocious proliferation. Cancer cells exhibit aberrant lipid metabolism for energy, bio-membrane composition, and signaling molecules required for proliferation, survival, invasion, metastasis, and therapeutic resistance [21]. Notably, lipid droplets can be found in GBMs but not in low-grade gliomas or normal brain tissues, and are closely related to the degree of malignancy [22,23].

Metabolic reprogramming is a primary cause of therapeutic resistance [24], and identifying mechanisms regulating lipid metabolism in GBM may reveal new paradigms to curb GBM growth and recurrence, and ultimately, a strategy to improve patient survival. SREBPs, including SREBP1a, SREBP1c and SREBP2, regulate the lipid level in cultured cells. SREBP1a and SREBP1c regulate the expression of genes involved in fatty acid synthesis, including ATP citrate lyase (ACLY), activating signal cointegrator 1 complex subunit 2 (ASCC2), fatty acid synthase (FASN, the key enzyme of de novo fatty acid synthesis), stearoyl-CoA desaturase (SCD) and fatty acid desaturase 1 (FADS1). While SREBP2 regulates cholesterol synthesis related genes, including 3-hydroxy-3-methylglutaryl-CoA reductase (HMGCR, the key enzyme of de novo cholesterol synthesis), squalene epoxidase (SQLE) and 3-hydroxy-3-methylglutaryl-CoA synthase 1(HMGCS1) [25,26]. The full-length SREBPs, also called precursors, located in endoplasmic reticulum (ER) membrane, bind to the SREBP-cleavage-activating protein (SCAP). When the sterol is depleted, the SCAP-SREBP complex can transfer to the Golgi, there SREBPs can be activated by proteolytic cleavage by two proteases. In addition, the mature SREBPs (N-terminal domain) can enter the nucleus and activate the downstream genes by binding the sterol regulatory element (SRE) in the promoters. When the level of sterol is high, the insulin-induced gene (Insig) will be recruited by SCAP, thus the Insig-SCAP-SREBP complex will be retained in the ER. When the SCAP is N-glycosylated by glucose, the protein can dissociate from Insig and transfer to Golgi, thus activating the SREBP1 [27,28,29]. SREBP1 and its encoding gene sterol regulatory element-binding transcription factor 1 (SREBF1) regulate lipid metabolic reprogramming, and their inhibition significantly induces GBM cell death [30,31,32,33], indicating that SREBP1 could be a promising target in GBM. Activation of the EGFR/AKT/SREBP1 signaling axis can promote GBM growth and survival by enhancing lipid metabolism [30]. The transcription factor liver X receptor (LXR) is a potential GBM therapeutic target due to its ability to promote sterol efflux transporters and decrease excess intracellular cholesterol [34]. SREBP inhibitors, such as fatostatin or betulin, show promising efficacy in inhibiting tumor growth [35,36].

This study aimed to investigate the effects of TAK901 in GBM in vitro and in vivo and explore its key downstream targets. We found that TAK901 significantly inhibited the stem cell-like phenotype of GBM cells and potently suppressed tumor growth in orthotopic GBM mouse models. Moreover, TAK901 suppressed the expression of the lipid metabolism-related gene SREBF1, whereas the exogenous expression of SREBP1 alleviated TAK901-mediated suppression of cell viability and apoptosis in GBM cells. This study suggests TAK901 as a potential therapeutic approach for GBM treatment.

## 2. Materials and Methods

### 2.1. Reagents and Antibodies

TAK901 (#T2709) was purchased from TargetMol (Shanghai, China). Primary antibodies were as follows: SREBP-1 (2A4) (#sc-13551, Santa Cruz Biotechnology, Santa Cruz, CA, USA), SREBP-2 (1C6) (#sc-13552, Santa Cruz Biotechnology, Santa Cruz, CA, USA), phospho-GSK-3β (Ser9) (D85E12) (#5558, CST, Danvers, MA, USA), β-actin (#20536-1-AP, Proteintech, Wuhan, China), Ki67 (Servicebio, Wuhan, China),histone H3 (#4499s, CST, Danvers, MA, USA), phospho-histone H3 (Ser10) (#53348T, CST, Danvers, MA, USA), Aurora A (#45-8900, Invitrogen, Carlsbad, CA, USA), Aurora B (#ab2254, abcam, Waltham, MA, USA) and c-Myc (#9402S, CST, Danvers, MA, USA). Secondary antibodies were as follows: anti-mouse IgG, HRP-linked antibody (#7076, CST, Danvers, MA, USA), anti-rabbit IgG, HRP-linked antibody (#7074, CST, Danvers, MA, USA).

### 2.2. Human GBM Cell Lines and Patient-Derived GSCs

The U87MG cell line was purchased from Cellcook (Guangzhou, China) and grown in Dulbecco’s Modified Eagle’s Medium (DMEM) (Gibco, Vacaville, CA, USA) supplemented with 10% FBS (ExCell Bio, Shanghai, China and 1% penicillin-streptomycin (Gibco, Vacaville, CA, USA).

The collection of surgical specimens from patients with GBM was approved by the Institutional Review Board of Xiangya Hospital of Central South University. The diagnosis of the GBM was confirmed by the neuropathologists. GSCs were derived as previously described [37]. Briefly, tumor tissues were cut into small pieces and digested with DNase I (Sigma, Burlington, MA, USA) and collagenase IV (Gibco, Vacaville, CA, USA). GSCs were grown in a GSC medium, which contained a neurobasal medium (Gibco, Vacaville, CA, USA) supplemented with 10 ng/mL basic fibroblast growth factor (bFGF) (PeproTech, Windsor, NJ, USA), 10 ng/mL epidermal growth factor (EGF) (PeproTech, Windsor, NJ, USA), 1 × GlutaMax (Invitrogen, Carlsbad, CA, USA), and 1% penicillin-streptomycin. GSC5 was derived from a female patient with GBM, with unmethylated MGMT, GFAP (+), Ki67 (40%+), P53 (−), IDH1 (−), H3K27M (−), Olig2 (+), EGFR (+), and ATRX (+).

GSC cultures would grow as spheroid cultures, especially under high cell density. The drug was added to GSC5 when seeding. For U87MG, the drug was added after 12 h for cell adherence.

### 2.3. Cell Viability Assays

A total of 1000 U87MG cells/well were seeded in 96-well plates in DMEM supplemented with 10% FBS. After 12 h, cells were treated with indicated concentrations of TAK901 for 24 h, 48 h and 72 h. For GSC5 cells, cells were seeded at a density of 1000 cells/well in 96-well low-attachment plates in a GSC medium. At the same time, GSC5 cells were treated with indicated concentrations of TAK901. Cell viability was measured using the CellTiter-Glo assay kit (Promega, Madison, WI, USA), according to the manufacturer’s protocol. For SREBP1 overexpression assay, U87MG cells were seeded in DMEM without FBS.

### 2.4. Cell Apoptosis Assays

U87MG cells (2 × 10^5^ cells/well) were seeded in 6-well plates. After 12 h, U87MG cells were treated with different concentrations of TAK901 (2.5, 5, and 10 μM) for 72 h. GSC5 cells (2 × 10^5^ cells/well) were seeded in 6-well low-attachment plates and at the same time incubated with 0.25 μM, 0.5 μM, and 1 μM of TAK901 for 72 h. All cells were collected and stained with annexin V-APC/PI according to the manufacturer’s protocol (Liankebio, Hangzhou, China). The cells were analyzed by flow cytometry (Beckman Coulter, Brea, CA, USA).

### 2.5. Live/Dead Assays

A total of 1000 U87MG cells/well were seeded in 96-well plates in DMEM supplemented with 10% FBS for 12 h. GSC5 cells (1000 cells/well) were seeded into 96-well low-attachment plates in a GSC medium. After 72 h of treatment with TAK901, calcein-AM, propidium iodide (PI), and Hoechst 33342 (Dojindo, Shanghai, China) were added to the plates. The signals of calcein-AM, PI, and Hoechst 33342 represented the live, dead, and total cells, respectively. Fluorescent images were captured, and the live and dead cell numbers were calculated by Operetta CLS (PerkinElmer, Waltham, MA, USA). For GSC5, the spheres, it is hard to calculate the cell number correctly, the relative sum area was used to quantify the assay by Operetta CLS (PerkinElmer, Waltham, MA, USA).

### 2.6. Cell Cycle Assays

U87MG cells (2 × 10^5^ cells/well) were seeded in 6-well plates for 12 h. TAK901 was then added at 0.31 μM for 72 h. GSC5 cells (2 × 10^5^ cells/well) were seeded and at the same time treated with 0.125 μM TAK901 for 72 h. Cells were fixed in cold 70% ethanol overnight at −20 °C and hydrated for 15 min before staining with a DNA staining solution (Liankebio, Hangzhou, China). The cells were analyzed using a flow cytometer (Beckman Coulter, Brea, CA, USA).

### 2.7. Transwell Assays

For the migration assay, 2 × 10^4^ U87MG cells/well treated with 2.5, 5, and 10 μM TAK901 were seeded in the top chamber of a Transwell chamber with 8 μm pores (Corning, Lowell, MA, USA). For the invasion assay, the top chamber of the Transwell was previously coated with 30 μL Matrigel matrix (1:8 diluted with PBS; Corning). After solidification, 3 × 10^4^ U87MG cells/well were seeded into the top chamber. The bottom chambers were loaded with 10% FBS-containing DMEM, while the top chambers were loaded without FBS. After 24 h, the cells that crossed the Transwell membrane were fixed with 4% paraformaldehyde (PFA) and stained with crystal violet (Beyotime, Shanghai, China). Images were captured, and the cell numbers within the same area were calculated using the LionheartFX automated imager and Gen5 software (BioTek, Winooski, VT, USA).

### 2.8. Three-Dimensional (3D) Cell Invasion Assays

A total of 4000 U87MG cells or GSC5 cells/well were seeded in a 384-well low-attachment sphere plate (Corning, Lowell, MA, USA). The plates were then centrifuged at 300 g for 5 min. After 24 h, TAK901 and Matrigel were added to the spheres. After 24 h, the sphere area was calculated using a LionheartFX automated imager and Gen5 software (BioTek, Winooski, VT, USA).

### 2.9. Neurosphere Assays

Five thousand U87MG cells or GSC5 cells/well were seeded in 24-well low-attachment plates (Corning, Lowell, MA, USA) and subjected to indicated treatments at the same time. After seven days, calcein-AM was added for better calculation. The plates were imaged in montage mode. All spheres were analyzed using the LionheartFX automated imager and Gen5 software (BioTek, Winooski, VT, USA).

### 2.10. Limiting Dilution Assay

U87MG or GSC5 cells (1, 5, 10, 20, or 50 cells/well) were plated in the 96-well low-attachment plate (Corning, Lowell, MA, USA) and treated with indicated concentrations of TAK901 at the same time. After 14 days, stem cell frequency was measured using LDA software [38] Accessed date: 3 August 2022 (https://bioinf.wehi.edu.au/software/elda/).

### 2.11. Lipid Measurements

The lipid-staining dye BODIPY 493/503 (GLPbio, Montclair, CA, USA) was used to stain neutral lipids. For U87MG cells, 1000 cells/well were plated in a 96-well plate for 12 h and then treated with 10 μM TAK901. For GSC5 cells, 2 × 10^5^ cells were plated in a 6-well low-attachment plate and treated with 0.2 μM TAK901 at the same time. After 72 h of treatment, a stock solution of BODIPY was added to the cells at a working concentration of 2 μM and an incubation time of 15 min. After washing with PBS, U87MG cells were double-stained with Hoechst 33342. Fluorescent images were captured using an Operetta CLS (PerkinElmer, Waltham, MA, USA). GSC5 cells were analyzed by flow cytometry (Beckman Coulter, Brea, CA, USA).

### 2.12. Western Blotting

U87MG cells (6 × 10^5^ cells) were seeded in a 60 mm dish for 12 h, then treated with various concentrations of TAK901. GSC5 cells (2 × 10^5^ cells) were seeded in a 6-well low-attachment plate, then treated with various concentrations of TAK901 at the same time. After incubation for 72 h, cell lysates were collected using a RIPA lysis buffer containing protease and phosphatase inhibitors. The protein concentration was measured using a BCA kit (GLPbio, Montclair, CA, USA). Proteins were added to the FuturePAGE 4–12% gel (ACE, Suzhou, China) and separated using an MOPS-SDS running buffer at 90 V for 90 min. The gels were transferred onto the PVDF membranes (Millipore, Burlington, MA, USA). Membranes with incubated with the ECL reagent (Millipore, Burlington, MA, USA), and chemiluminescence signals were captured using a ChemiDoc imaging system (Bio-Rad, Hercules, CA, USA).

### 2.13. Gene Overexpression by Lentivirus

Overexpression vectors for mature SREBP1 (2–490 amino acid residues) and mature SREBP2 (2–485 amino acid residues) were constructed using pTSBOE-puro lentiviral vector. The primers used are listed in Table 1. Plasmids were generated using the ClonExpress II One Step Cloning Kit (Vazyme, Nanjing, China). psPAX2 and pMD2.G plasmids were used as lentiviral packaging vectors, and 293T cells were used to produce lentiviruses. Polybrene (10 μg/mL) was used for transfection. After two days of transfection, transfected U87MG cells were selected by a FACSAria III cell sorter (BD Bioscience, La Jolla, CA, USA) and maintained in culture medium with 5 μg/mL puromycin. The primers are listed in Table 1.

### 2.14. Reverse Transcription-Quantitative Real-Time Polymerase Chain Reaction (RT-qPCR) Assays

U87MG cells (6 × 10^5^ cells) were seeded in a 60 mm dish for 12 h, then treated with 10 μM TAK901. GSC5 cells (2 × 10^5^ cells) were seeded in a 6-well low-attachment plate, then treated with 1 μM TAK901 at the same time. After treatment for 72 h, RNA was extracted using a FastPure Cell/Tissue Total RNA Isolation Kit (Vazyme, Nanjing, China). cDNAs were synthesized using HiScript II Q RT SuperMix (Vazyme). For quantitative real-time PCR, reactions were performed using ChamQ Universal SYBR qPCR Master Mix (Vazyme, Nanjing, China). The primers used are listed in Table 2.

### 2.15. In Vivo Orthotopic Tumor Mouse Model

U87MG-luc cells (2 × 10^5^) were implanted intracranially in 6–8 week-old female BALB/c nude mice. Using a stereotaxic apparatus (RWD Instruments, Shenzhen, China), 5 μL of a U87MG-luc cell suspension was slowly injected (1.5 μL/min) into the brain. The location was 1 mm anterior, 2 mm lateral to the bregma, and 3 mm deep from the cortical surface. After 7 days, 5 μL of 2 mM TAK901 or vehicle (six mice per group) was injected via a stereotaxic injection. The injection frequency was twice weekly. Tumor growth was monitored using an IVIS imaging system (PerkinElmer, Waltham, MA, USA) before drug injection. Mouse body weight was measured at the same time. Mice were sacrificed by CO_2_ euthanasia on day 17 after IVS imaging. The brains were fixed with 4% PFA, and the tumor sections were subjected to hematoxylin and eosin and Ki67 immunohistochemical staining. Images were captured using a LionheartFX automated imager (BioTek, Winooski, VT, USA). The tumor volumes and Ki67 positive cell numbers were analyzed using Gen5 imaging software (BioTek, Winooski, VT, USA). Tumor volumes were calculated by the following formula: volume (mm^3^) = (length × width × width) × 0.52 [39].

Mice were housed in an SPF animal room and maintained on a 12-h light-dark cycle in the RuiYe laboratory with autoclaved food, bedding and water. All operations were conducted in accordance with the animal care regulations.

### 2.16. RNA-Sequencing

U87MG cells (6 × 10^5^ cells) were seeded in a 60 mm dish for 12 h, then treated with 0.2 μM TAK901. GSC5 cells (2 × 10^5^ cells) were seeded in a 6-well low-attachment plate, then treated with 0.1 μM TAK901 at the same time. After treatment for 72 h, RNA was isolated using the RNeasy mini kit and subjected to RNA-seq on an Illumina Hi-seq 3000 system using a genomics core facility protocol. Differentially expressed genes (DEGs) were determined using DESeq2 software with the criteria of padj <  0.05 and abs(log2(fold change)) ≥ 0.5. Biological significance of the genes was determined using Gene Ontology (GO) enrichment and gene set enrichment analysis (GSEA) software. Heatmaps, volcano plots, and bubble plots were drawn using R to illustrate the results.

### 2.17. Data Analysis

GraphPad Prism8 was used to perform the statistical analysis. Data significance was analyzed using the ordinary one-way analysis of variance (ANOVA) or *t*-tests. Statistical significance was set as *p* < 0.05 (*), *p* < 0.001 (**), *p* < 0.001 (***), *p* < 0.0001 (****).

## 3. Results

### 3.1. TAK901 Reduces Cell Viability and Induces Apoptosis, Cell Cycle Arrest, and Polyploid Formation in GBM Cells

**Figure 1:**
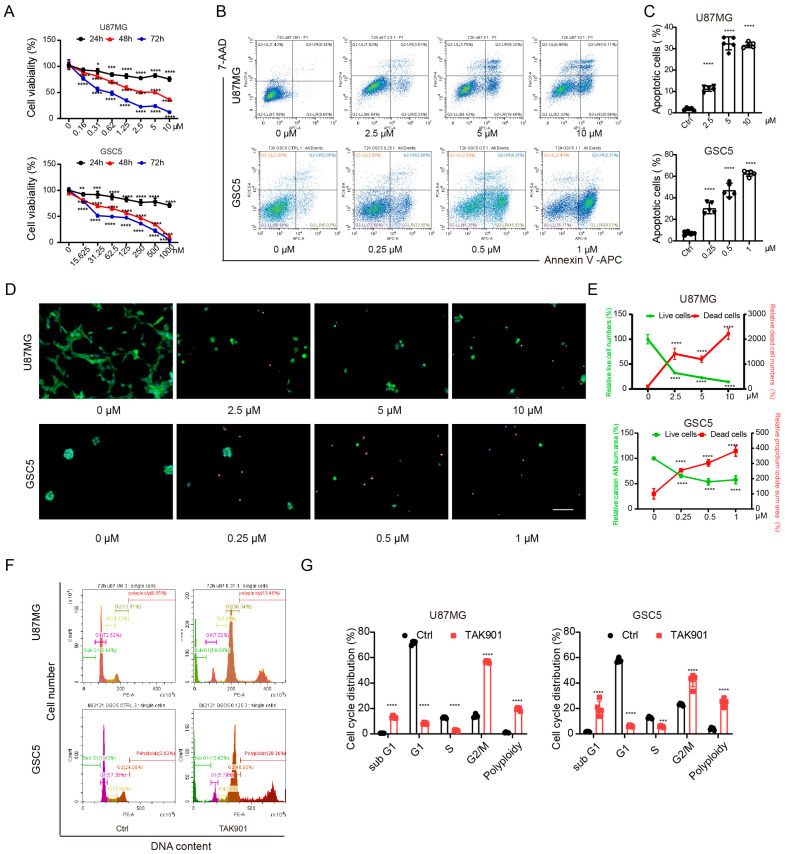
TAK901 inhibits the proliferation of glioblastoma (GBM) cells and causes cell cycle arrest and polyploid. (**A**) U87MG and GSC5 cells were treated with increasing concentrations of TAK901 for 24 h, 48 h and 72 h. The cell viability was analyzed. CellTiter-Glo assays were performed in triplicate, and representative results are shown (*n* = 5). (**B**) U87MG and GSC5 cells were treated with increasing concentrations of TAK901 for 72 h. The cells were subjected to Annexin V and 7-AAD staining and analyzed by flow cytometry. (**C**) Quantification of the total apoptotic cells (*n* = 5). (**D**) U87MG and GSC5 cells were treated with the indicated concentrations of TAK901 for 72 h. Calcein-AM and propidium iodine were used to identify live and dead cells, respectively. The images were captured by Operetta high content screening. Scale bar, 100 μm. (**E**) Quantification of live and dead cells in U87MG and GSC5 (*n* = 5). (**F**) Cell cycle assays through PI staining. U87MG and GSC5 cells were treated with low concentration of TAK901 for 72 h. (**G**) Quantification of the cell cycle assays in U87MG and GSC5 (*n* = 5). Significance was determined by one-way ANOVA with Dunnett’s multiple comparison adjustment (**A**,**C**,**E**) or unpaired and two-tailed Student’s *t*-test (**G**). * *p* < 0.05, ** *p* < 0.001, *** *p* < 0.001, **** *p* < 0.0001.

To investigate the antitumor efficacy of TAK901 in GBM, we treated both the classical U87MG GBM cell line and patient-derived glioma stem cell line GSC5 with increasing concentrations of TAK901. The results of the cell viability assay showed that TAK901 significantly suppressed the proliferation of GBM cells (Figure 1A). Additionally, TAK901 considerably increased the proportion of apoptotic cells in both U87MG and GSC5 cell lines in a dose-dependent manner (Figure 1B,C). We further validated this finding using live/dead assays, which revealed that TAK901 increased the percentage of dead U87MG and GSC5 cells and decreased the percentage of live U87MG and GSC5 cells in a similar manner (Figure 1D,E). TAK901 is a known Aurora kinase inhibitor that is tightly correlated with the cell cycle process and polyploid formation. Therefore, we further performed cell cycle assays in GBM cells with or without low concentration TAK901. We found that TAK901-treated cells had a higher percentage of tetraploids and considerably fewer diploids, indicating a remarkable cell cycle arrest. The percentage of sub G1 cells increased and the polyploid cells appeared. (Figure 1F,G). Collectively, these findings suggest that TAK901 reduces the viability of GBM cells and induces apoptosis, cell cycle arrest and polyploid formation.

### 3.2. TAK901 Suppresses the Migration, Invasion, and Stemness of GBM Cells

**Figure 2:**
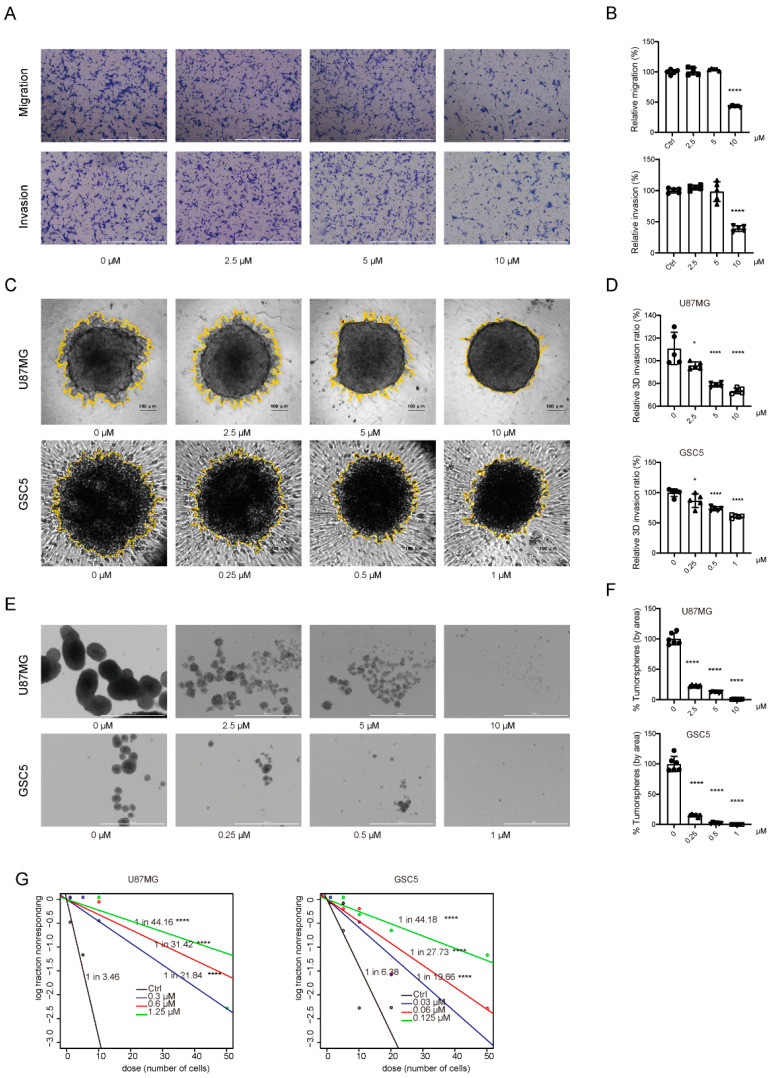
TAK901 suppresses the migration and invasion of GBM cells and the stemness of glioma stem cells. (**A**) Representative images of migration and invasion assays of U87MG cells treated with indicated concentrations of TAK901 for 24 h using 24-well Transwell inserts (*n* = 5). Scale bar, 1000 μm. (**B**) Quantification of the numbers of migrating and invading cells using Gen5 software. (**C**) Representative images of 3D Matrigel invasion assay of U87MG and GSC5 cells treated with different concentrations of TAK901 for 24 h (*n* = 5). Scale bar, 100 μm. (**D**) The yellow rings in the pictures of the 3D invasion were drawn by Gen5 software, which quantified the area of the sphere. The relative invasion ratio is shown as a percentage of the control groups. (**E**) U87MG cells and GSC5 cells were grown in 24-well low-attachment plates and treated with increasing concentrations of TAK901 for 7 days to form tumor spheres (*n* = 5). Scale bar, 1000 μm. (**F**) The numbers of U87MG and GSC5 tumor spheres were calculated by Gen5 software. (**G**) The stem cell frequency of U87MG and GSC5 cells. Limitation dilution assays of U87MG and GSC5 cells were performed by treatment with indicated concentrations of TAK901 or vehicle for 14 days (*n* = 10). Significance was determined by one-way ANOVA with Dunnett’s multiple comparison adjustment (**B**,**D**,**F**). * *p* < 0.05, **** *p* < 0.0001.

Tumor invasiveness, therapeutic resistance, and recurrence are highly associated with poor prognosis of patients with GBM. Therefore, we investigated the effect of TAK901 on the migration, invasion, and stemness of GBM cells. The Transwell assay showed that the TAK901-treated group had fewer cells migrating through the chambers, indicating that the migration and invasion ability of U87MG was impaired after treatment with TAK901 (Figure 2A,B). To validate this result, we performed a 3D Matrigel invasion assay for both U87MG and GSC5 cells, and the results demonstrated that compared to treatment with a vehicle, treatment with TAK901 decreased the 3D invasion ratio in a dose-dependent manner (Figure 2C,D).

GSCs (or TICs) exhibit stem cell-like properties and are profoundly involved in the therapeutic resistance and recurrence of GBM [7,8]. Therefore, we investigated the effect of TAK901 on the stemness of GBM cells. Self-renewal and neurosphere formation assays revealed that treatment with TAK901 significantly reduced the sphere area and stem cell frequency in both U87MG and GSC5 cells (Figure 2E–G). Therefore, these results suggest that TAK901 can reduce GSC invasiveness and stemness in vitro.

### 3.3. TAK901 Inhibits GBM Growth in Orthotopic Xenograft Mouse Models

**Figure 3:**
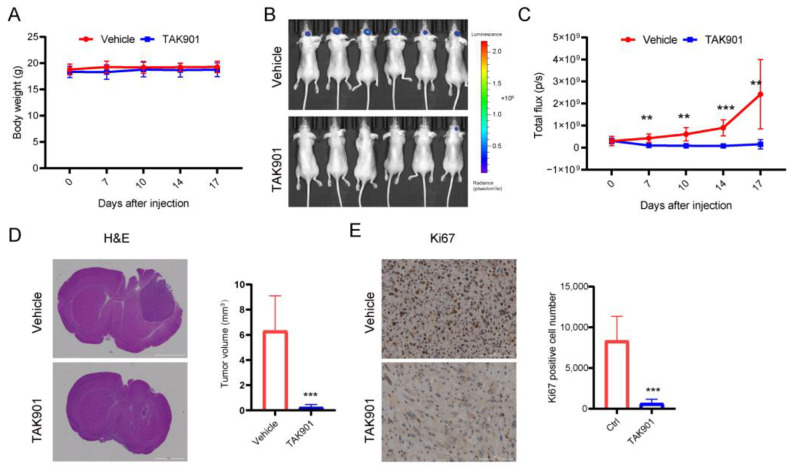
TAK901 inhibits tumor growth in vivo. U87MG-LUC cells were implanted into the brain of BALB/c nude mice. After 7 days, the tumors were established. The mice were randomized to the vehicle or the TAK901 treated group. *n* = 6. (**A**) Changes in the body weight of mice over time are shown. The body weight was measured twice per week after injection. (**B**) IVIS images were taken on day 17 after injection. (**C**) Quantification of the bioluminescence signal in (**B**). (**D**) Hematoxylin and eosin (H&E) staining of tumor sections. Mice were sacrificed on day 17. Brains were fixed and the tumor sections were stained by H&E. The tumor volume was quantified based on the H&E pictures. Scale bar, 2000 μm. (**E**) Ki67 staining of tumor sections. The brains were fixed, the tumor sections were stained by Ki67, and the Ki67-positive cells were quantified. All analyses were performed using Gen5 software (BioTek, USA). Scale bar, 100 μm. Significance was determined by unpaired and two-tailed Student’s *t*-test (**C**–**E**). ** *p* < 0.001, *** *p* < 0.001.

Our in vitro studies have shown that, at the cellular level, TAK901 can suppress GBM proliferation, invasion, and stemness, and induce apoptosis and cell cycle arrest. To further determine whether TAK901 can inhibit GBM growth in vivo, we injected U87MG-luc cells into the right cerebrum of BALB/c nude mice and then treated them with stereotaxic injections of vehicle or TAK901 (six mice per group). We found that TAK901 significantly decreased tumor growth without affecting the body weight of tumor-bearing mice (Figure 3A–C). Furthermore, immunohistochemical staining of tumor sections showed that both tumor size and proliferation marker Ki67 were reduced in the TAK901-treated group (Figure 3D,E). These results suggest that TAK901 significantly inhibits GBM growth in an orthotopic xenograft mouse model.

### 3.4. TAK901 Reduces the Expression of Genes Involved in Lipid Metabolism

**Figure 4:**
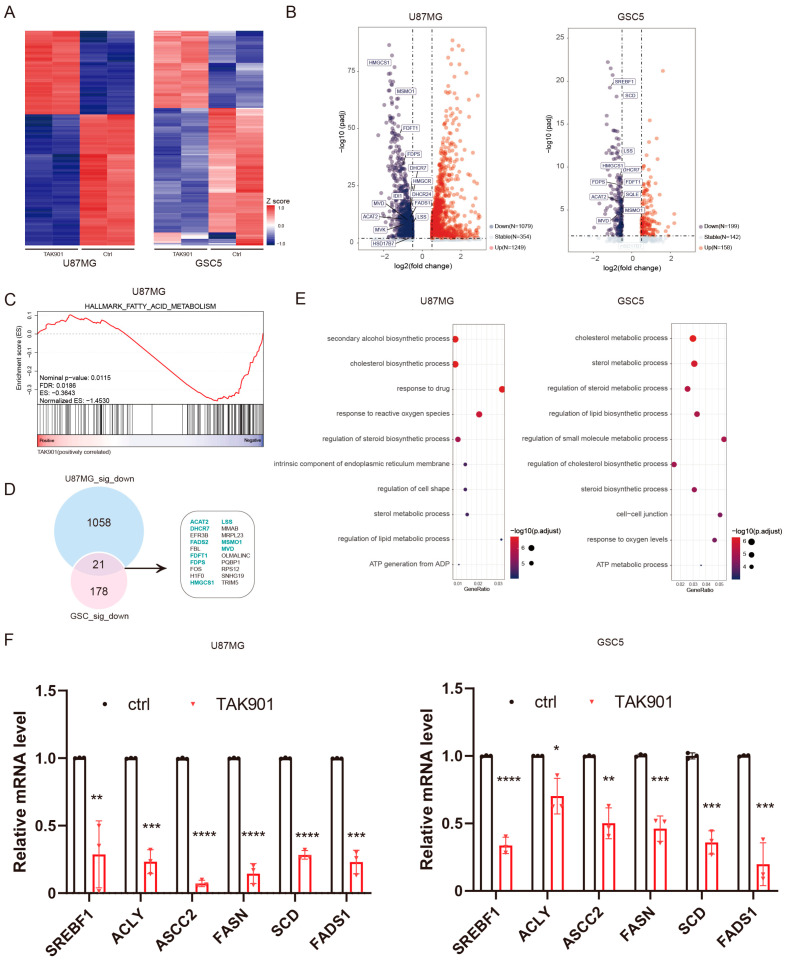
TAK901 suppresses the expression of genes related to lipid metabolism. U87MG cells were treated with 0.2 μM TAK901 or vehicle for 72 h. GSC5 cells were treated with 0.1 μM TAK901 or vehicle for 72 h. The cells were subjected to transcriptomic analysis. *n* = 2. (**A**) Heatmaps showing the robustness of the RNA-seq data for U87MG (**left**) and GSC5 (**right**) cells. Plot showing the top 200 commonly regulated (padj < 0.05 and log2FC ≤ −0.5 or log2FC ≥ 0.5) genes of U87MG and GSC5 cells. (**B**) Volcano plot of the expression of genes in TAK901-treated U87MG (**left**) and GSC5 (**right**) cells. Plot showing the commonly regulated (padj < 0.05 and log2FC ≤ −0.5 or log2FC ≥ 0.5) genes of U87MG and GSC5 cells. Red: significantly upregulated genes; blue: significantly downregulated genes, gray: non-affected genes. Some genes related to the lipid metabolism pathway are labeled. FC: fold change. (**C**) Gene set enrichment analysis (GSEA) plot depicted the DEGs identified between control and TAK901 treated group in U87MG. Fatty acids metabolism is shown. (**D**) Overlap gene set of significantly downregulated (padj < 0.05 and log2FC ≤ −0.5) genes of U87MG (**up**) and GSC5 (**down**). Lipid metabolism-related genes are highlighted. (**E**) Gene Ontology enrichment analysis of downregulated genes in U87MG (**left**) and GSC5 (**right**) cells. Dots on the graph indicate enriched pathways. Most pathways are related to lipid metabolism. (**F**) U87MG cells were treated with 10 μM TAK901 or vehicle for 72 h. GSC5 cells were treated with 1 μM TAK901 or vehicle for 72 h. Expression of genes related to fatty acid metabolism in U87MG (**left**) and GSC5 (**right**) cells were measured by RT-qPCR. *n* = 3 (biological replicates). Significance was determined by unpaired and two-tailed Student’s *t*-tests (**F**). * *p* < 0.05, ** *p* < 0.001, *** *p* < 0.001, **** *p* < 0.0001.

To investigate the specific molecular mechanism of the antitumoral effect of TAK901 in GBM, we treated U87MG and GSC5 cells with either vehicle or TAK901 and examined global transcriptional changes by RNA-sequencing. A correlation analysis of the samples showed the good robustness of the RNA-seq data. Duplicates in the same group had good repeatability but differed significantly from samples from the other groups (Supplementary Figure S1A). The heatmap of the DEGs indicated that TAK901 profoundly altered the transcriptional activities of GBM cells, with a large number of DEGs identified (Figure 4A,B). Importantly, GSEA results indicated that genes altered by TAK901 showed negative enrichment with the gene sets of fatty acid metabolism (Figure 4C) and cholesterol homeostasis pathways (Supplementary Figure S1B). Notably, further analysis of the DEGs revealed that many lipid metabolism pathway genes were significantly downregulated in both the U87MG and GSC5 cell lines, as shown in the Venn plot and labeled in the volcano plot (Figure 4B,D). Consistently, GO enrichment analysis of significantly downregulated genes showed that many lipid metabolism-related pathways were inhibited by TAK901 (Figure 4E). Gene correlation analysis of the RNA-seq data also revealed that the expression of many lipid metabolism pathway-related genes was positively correlated with each other (Supplementary Figure S1C). TAK901 inhibited the expression of genes regarding fatty acid and cholesterol synthesis (supplementary Figure S1D). Moreover, the inhibition of some identified lipid metabolism genes in both cell lines were validated using RT-qPCR assays, including SREBF1, ACLY, ASCC2, FASN, SCD, and FADS1 (Figure 4F). These results suggest that the effect of TAK901 on GBM inhibition may involve the attenuation of lipid metabolism.

### 3.5. TAK901 Decreases Neutral Lipids and Inhibits GBM Growth through Sterol Regulatory Element-Binding Protein 1 (SREBP1)

**Figure 5:**
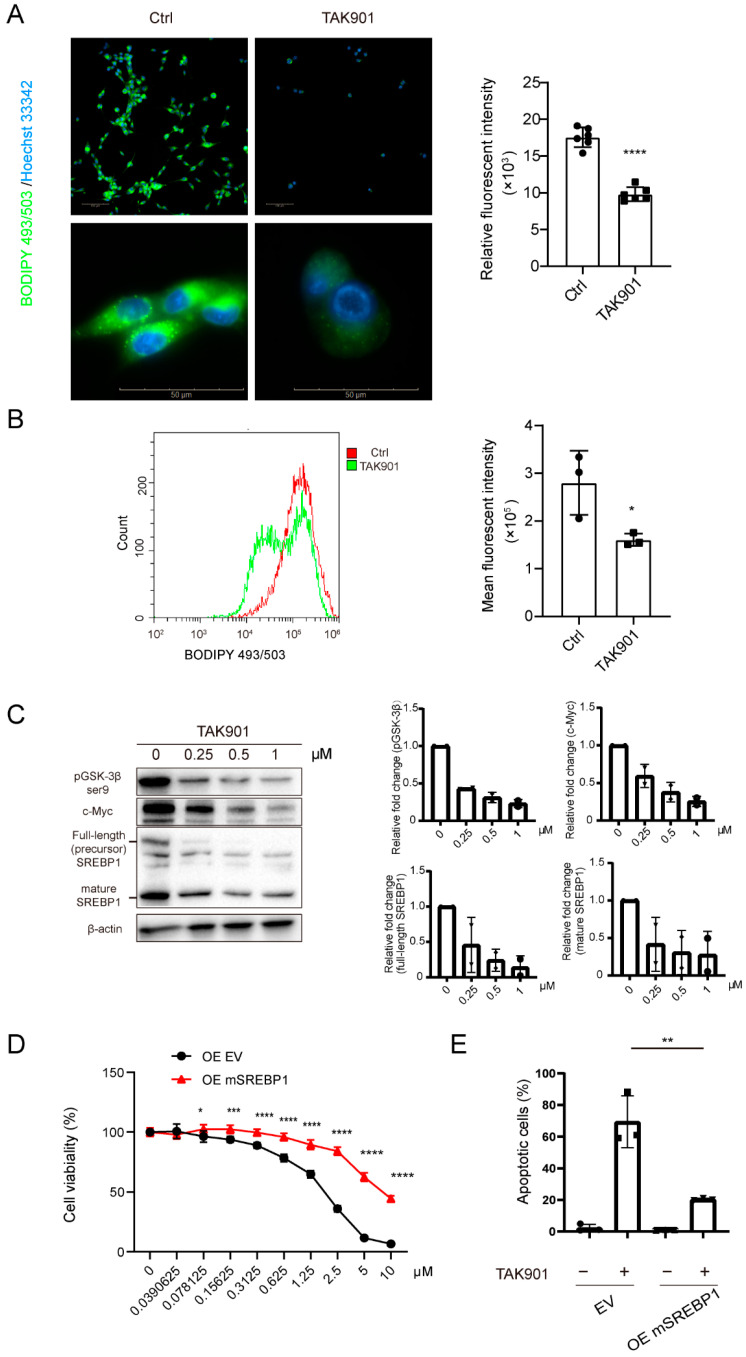
TAK901 inhibits tumor growth through SREBP1. (**A**) BODIPY 493/503 staining of U87MG cells. U87MG cells were treated with 10 μM TAK901 or vehicle for 72 h. The cells were labeled with the green, fluorescent dye BODIPY 493/503 to stain the neutral lipids. The images were captured by Operetta high content screening. The mean relative fluorescence was calculated using Harmony software (*n* = 6). Scale bar, 100 μm (**top**), 50μm (**bottom**). (**B**) Histogram of BODIPY 493/503 signals in GSC5 cells. GSC5 cells were treated with 0.2 μM TAK901 or vehicle for 72 h. After BODIPY 493/503 staining, the cells were subjected to flow cytometry. Mean fluoresce intensity (MFI) was calculated by Flowjo (*n* = 3). (**C**) Western blot analysis of the protein expression of full-length SREBP1, mature SREBP1, pGSK3β and c-Myc in GSC5 cells (**left**). GSC5 cells were treated with the indicated concentrations of TAK901 or vehicle for 72 h. Quantification of each band was shown (**right**). (**D**) SREBP1 overexpression. U87MG cells were transfected with mSREBF1 overexpression plasmids. U87MG OE cells were treated with indicated concentrations of TAK901 or vehicle for 72 h without serum. The cell viability was calculated (*n* = 6). (**E**) Flow cytometry analysis of apoptotic U87MG OE cells in (**D**). Cells were treated with 10 μM TAK901 (*n* = 3). Significance was determined by unpaired and two-tailed Student’s *t*-test (**A**,**B**,**D**,**E**). * *p* < 0.05, ** *p* < 0.001, *** *p* < 0.001, **** *p* < 0.0001.

The RNA-seq results showed that TAK901 plays a critical role in lipid metabolism; therefore, we investigated the effect of TAK901 on the lipid content of GBM cells. BODIPY 493/503 staining showed that neutral lipids in GBM cells decreased significantly after treatment with TAK901 (Figure 5A,B), indicating that TAK901 hinders the lipid metabolism of GBM. Previous studies have found that SREBP1 is a key modulator of lipid metabolism and has a significant tumor-promoting effect in multiple cancers, including GBM [40,41]. Consistently, our RNA-seq data and RT-qPCR results also suggested that SREBF1 expression levels were remarkably reduced in TAK901-treated GBM cells. Therefore, we hypothesized that SREBP1 may be involved in the TAK901-mediated anti-GBM effect. To test this hypothesis, we evaluated SREBP1 expression levels in GBM cells treated with or without TAK901 using Western blotting. The results showed that TAK901 significantly downregulated the expression levels of both full-length SREBP1, mature SREBP1, p-GSK3βser9 and c-Myc in GBM cells (Figure 5C, Supplementary Figure S2A). To determine the role of SREBP1 in the TAK901-mediated anti-GBM effect, we transfected U87MG cells with an exogenous mSREBP1 plasmid to establish the SREBP1 overexpression cell line. Both the wild-type cell line and the SREBP1 overexpression cell line were treated with vehicle or serial doses of TAK901. The CellTiter-Glo assay results showed that SREBP1 overexpression significantly alleviated TAK901-mediated tumor inhibition (Figure 5D). Consistently, flow cytometry experiments showed that compared to the wild-type group, SREBP1 overexpression significantly rescued TAK901-induced apoptosis (Figure 5E). Notably, since SREBP1 and SREBP2 usually play similar roles in lipid metabolism, we also established the SREBP2 overexpression U87MG cell line and performed experiments similar to those of SREBP1 overexpression. The results suggest that SREBP2 also alleviates TAK901-mediated tumor inhibition and apoptosis in GBM (Supplementary Figure S2B,C). However, based on the RNA-seq data and RT-qPCR results, the difference in SREBF2 expression between the vehicle and TAK901 groups was not significant. Therefore, we assumed that its alteration is less likely to be the mechanism of action of TAK901. Overall, these results demonstrate that TAK901 hinders lipid metabolism in GBM and that SREBP1 may be a key downstream target.

To confirm the on target effect of TAK901, we performed the Western blot analysis of phospho-histone H3 (Ser10). After treatment with increased concentration of TAK901, the protein expression level of phospho-histone H3 (Ser10) was dose-dependently decreased, while the protein expression level of total histone H3 was not changed (Supplementary Figure S3A,B). This confirmed that the TAK901 concentration used in our study had no off target effect. The protein expression level of Aurora A and B in the two cell lines were measured using Western blotting assays. The protein expression of Aurora A and B are both higher in GSC5 (Supplementary Figure S3C).

## 4. Discussion

The antitumor effect of TAK901 has been documented in several human malignancies, including ovarian cancer and leukemia [16]. However, considerable knowledge gaps remain on the specific role of TAK901 in GBM, its mechanism of action, and its effect on lipid metabolism. Our findings address these challenges by establishing that (1) TAK901 reduces the viability, self-renewal, migration, invasion, and induces apoptosis and cell cycle arrest in both GBM and GSC cells; (2) TAK901 suppresses GBM growth in orthotopic mouse models; (3) TAK901 suppresses the expression of lipid metabolism pathway-related genes and reduces intracellular lipid droplets in GBM; and (4) TAK901 inhibits SREBP1 expression in GBM cells, whose overexpression attenuates the TAK901-mediated suppression of cell viability and apoptosis.

TAK901 regulates various oncogenic processes in multiple tumors. It induces cell cycle arrest and polyploidy in PC3 and HL60 cells and inhibits proliferation in many extracranial tumors, including ovarian, breast, and colorectal cancers [16]. Consistent with these findings, our results suggest that TAK901 suppresses cell viability and induces cell cycle arrest in GBM and GSC cells. GSC cells rely on polyunsaturated fatty acid synthesis to support EGFR signaling [19]. Therefore, it seems that the GSC cells, rather than GBM cells, were more sensitive to TAK901. Meanwhile, the protein expression of Aurora A and B were higher in GSC5 cells, which may also contribute to the drug sensitivity. The role of TAK901 in other carcinogenic processes such as invasion and metastasis are not well-documented. Several other Aurora kinase inhibitors have been extensively explored for the management of various tumors. The kinase Aurora inhibitor alisertib inhibits tumor metastasis by suppressing the epithelial-to-mesenchymal transition in Erα (+) breast cancer cells [42]. The selective Aurora B kinase inhibitor AZD1152 induces apoptosis in colon, lung and hematological tumors [43,44]. Inhibition of Aurora kinase activity by VX680 and valproic acid not only reduces neurosphere formation but also induces neuronal differentiation of GSC cells [45]. Our results consistently showed that TAK901 reduces self-renewal, migration, and invasion, induced apoptosis, and suppressed tumor growth in vivo.

In our study, the transcriptional analysis of TAK901-regulated genes revealed the downregulation of lipid metabolism pathway genes, which was further confirmed by RT-qPCR. Furthermore, a cellular lipid determination assay suggested that TAK901 reduced the neutral lipid content in GBM cells. This evidence suggests that alterations in lipid metabolism play a vital role in TAK901-mediated antitumor effects in GBM. In this study, TAK901 significantly downregulated SREBP1 in both differentiated GBM and GSC cells. Importantly, SREBP1 overexpression with exogenous plasmids remarkably attenuated the TAK901-mediated anti-GBM effects.

The aurora A kinase inhibitor alisertib has been reported to reduce the c-Myc protein. Aurora A kinase acted as a chaperonin of c-Myc, and protected c-Myc away from proteasomal degradation [46]. In our study, we found that TAK901 can decrease the expression of c-Myc. c-Myc induced the mRNA expression level of SREBP1, which regulates fatty acid synthesis [47]. Aurora A kinase phosphorylated the GSK3β protein at Ser 9 [46,48]. Phosphorylation of Ser-9 in GSK3β was reported to reduce the kinase activity of GSK3β. GSK3β phosphorylated the T426 and/or S430 in SREBP1, and induced the Fbw7-mediated degradation [49]. In our study, Western blot analysis revealed that TAK901 decreased the expression of phosphorylated GSK3β. Taken together, we speculate that TAK901 reduced the phosphorylation of GSK3β, making it more active, resulting in the degradation of SREBP1 and therefore regulating the lipid metabolism. TAK901 also controlled the mRNA expression of SREBP1 by reducing the c-Myc protein.

## 5. Conclusions

In summary, our results establish the potential of TAK901 to suppress GBM in vitro and in vivo by downregulating SREBP1 and attenuating lipid metabolism pathways. Thus, TAK901 may represent a novel therapeutic approach in the treatment of GBM.

## Figures and Tables

**Table 1:** PCR primers.

Gene Name	Primers
mSREBP1 F	attctagagctagcgaattcATGGACGAGCCACCCTTCAG
mSREBP1 R	catccttgtagtcggatccctaCAGGGCCAGGCGGGAGCG
mSREBP2 F	gaagattctagagctagcgaattcATGGACGACAGCGGC
mSREBP2 R	tcgtcatccttgtagtcggatccCAGAAGAATCCGTGAGCGGT

**Table 2:** RT-qPCR primers.

Gene Name	Primers
SREBF1 F	GCCCCTGTAACGACCACTG
SREBF1 R	CAGCGAGTCTGCCTTGATG
SCD F	TCTAGCTCCTATACCACCACCA
SCD R	TCGTCTCCAACTTATCTCCTCC
ACLY F	TGCTCTGAAATTGCCTTGG
ACLY R	CGGACTTCGGCAGAGGTAG
FASN F	CAGGCACACACGATGGAC
FASN R	CGGAGTGAATCTGGGTTGAT
ACSS2 F	AAAGGAGCAACTACCAACATCTG
ACSS2 R	GCTGAACTGACACACTTGGAC
FADS1 F	CTACCCCGCGCTACTTCAC
FADS1 R	CGGTCGATCACTAGCCACC
ACTB F	CATGTACGTTGCTATCCAGGC
ACTB R	CTCCTTAATGTCACGCACGAT

## Data Availability

The data presented in this study are available on request from the corresponding author.

## Supplementary Materials

The following supporting information can be downloaded at: https://www.mdpi.com/article/10.3390/cancers14235805/s1, Figure S1: GSEA of TAK901 treated cells; Figure S2: Western blotting of SREBP1 in U87MG and cell viability of U87MG overexpress SREBP2; Figure S3: TAK901 suppress histone H3 phosphorylation in GBM cells.
