# Supplementary material for: The Aurora Kinase Inhibitor TAK901 Inhibits Glioblastoma Growth by Blocking SREBP1-Mediated Lipid Metabolism
Source: Cancers (Basel). 2022 Nov 25;14(23):5805. doi: 10.3390/cancers14235805 (PMC9737940; doi:10.3390/cancers14235805)
Supplement: Supplementary file 1 [file cancers-14-05805-s001.zip › cancers-2014174-supplementary.pdf]

Supplementary Figure S1. GSEA and heatmap of gene expression by RNA-seq in U87MG and GSC5 cells after TAK901 treatment. TAK901 suppresses the expression of genes related to lipid metabolism. U87MG cells were treated with 0.2 μM TAK901 or vehicle for 72h. GSC5 cells were treated with 0.1 μM TAK901 or vehicle for 72h. The cells were subjected to transcriptomic analysis. n=2.

(A) Heatmaps showing the robustness of the RNA-seq data in U87MG and GSC5 cells.

(B) Gene set enrichment analysis plot of U87MG (top) and GSC5 (bottom) treated with TAK901 (left) versus control (right). Cholesterol homeostasis is shown.

(C) Correlation of the expression of lipid metabolism-related genes in the RNA-seq data.

(D) Heat map of gene expression analyzed by RNA-seq in U87MG and GSC5 cells after TAK901 treatment for 72h.

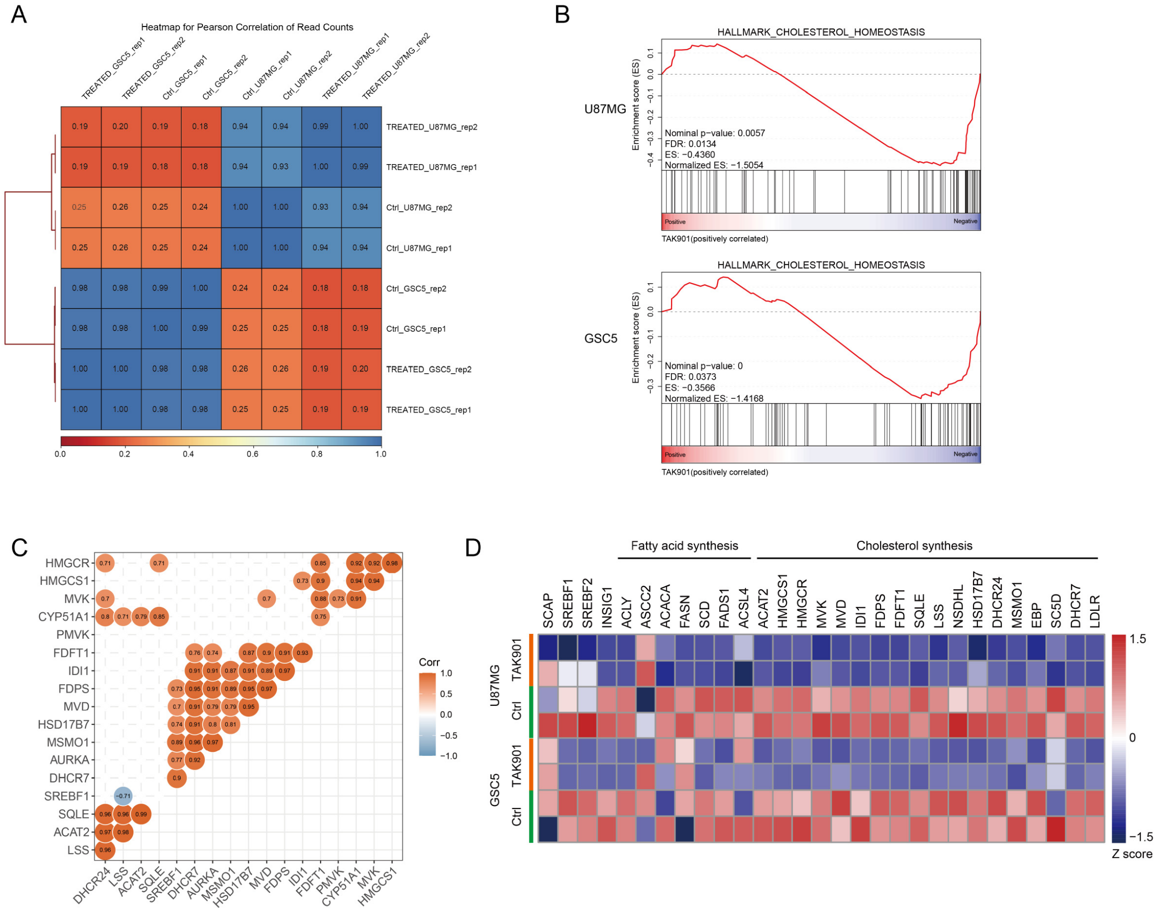

Supplementary Figure S2. Western blotting of SREBP1 in U87MG and cell viability of U87MG overexpress SREBP2. U87MG cells were treated with different concentration of TAK901 or vehicle for 72h.

(A) Western blot analysis of the protein expression of full-length SREBP1, mature SREBP1, full-length SREBP1, mature SREBP1, pGSK3β and c-Myc in U87MG cells (left). U87MG cells were treated with different concentration of TAK901 or vehicle for 72h. Quantification of each band was shown (right).

(B) U87MG cells were transfected with a plasmid overexpressing mSREBF2. The cell viability was calculated after treatment with different concentrations of TAK901 (n=6).
(C) Flow cytometry analysis of apoptotic U87MG cells. Cells were treated with 10 µM of TAK901 (n=3).
Significance was determined by unpaired and two-tailed Student's t-test (B, C). *P<0.05, **P<0.001, ***P<0.001, ****P<0.0001.

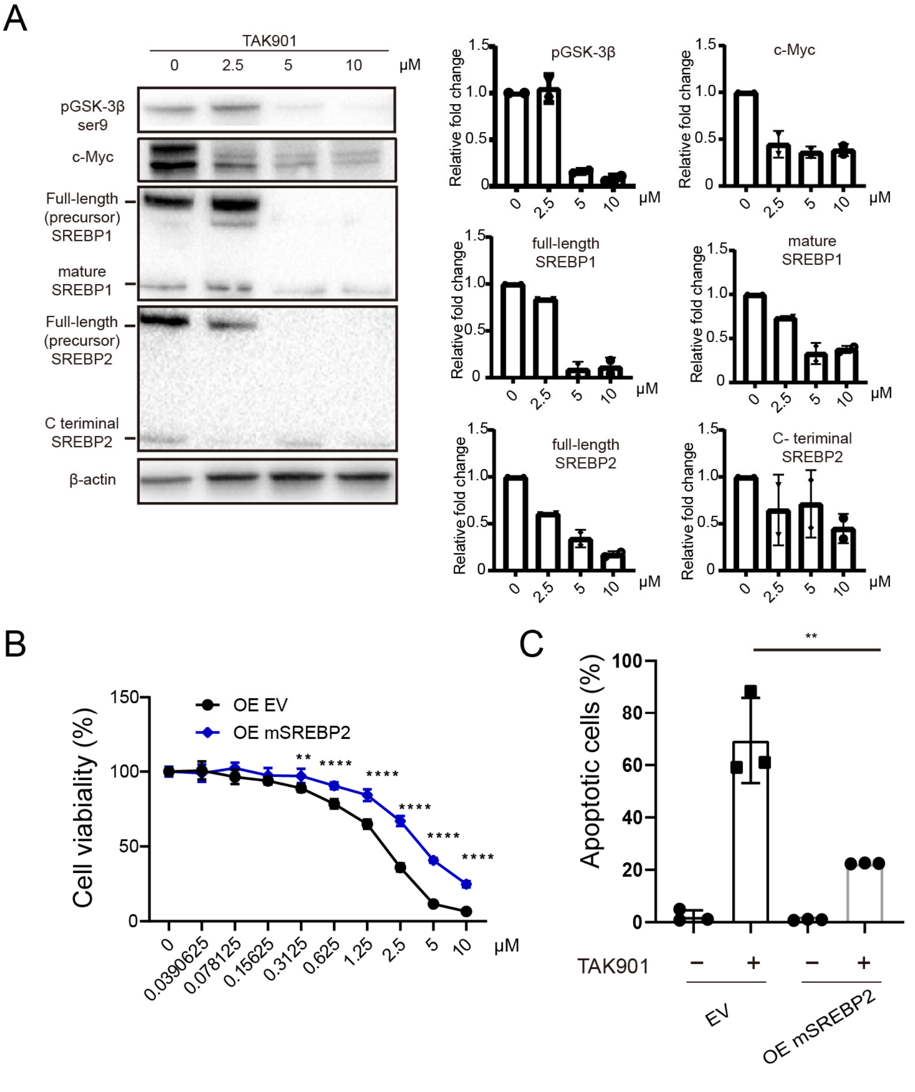

Supplementary Figure S3. TAK901 suppress histone H3 phosphorylation in GBM cells.
(A) Western blot analysis of the protein expression of phospho-histone H3 (Ser10) and histone H3 in U87MG cells. U87MG cells were treated with different concentration of TAK901 or vehicle for 72h. Quantification of each band was shown (right).
(B) Western blot analysis of the protein expression of phospho-histone H3 (Ser10) and histone H3 in GSC5 cells. GSC5 cells were treated with different concentration of TAK901 or vehicle for 72h. Quantification of each band was shown (right).
(C) Western blot analysis of the protein expression of Aurora A and B in U87MG and GSC5 cells.

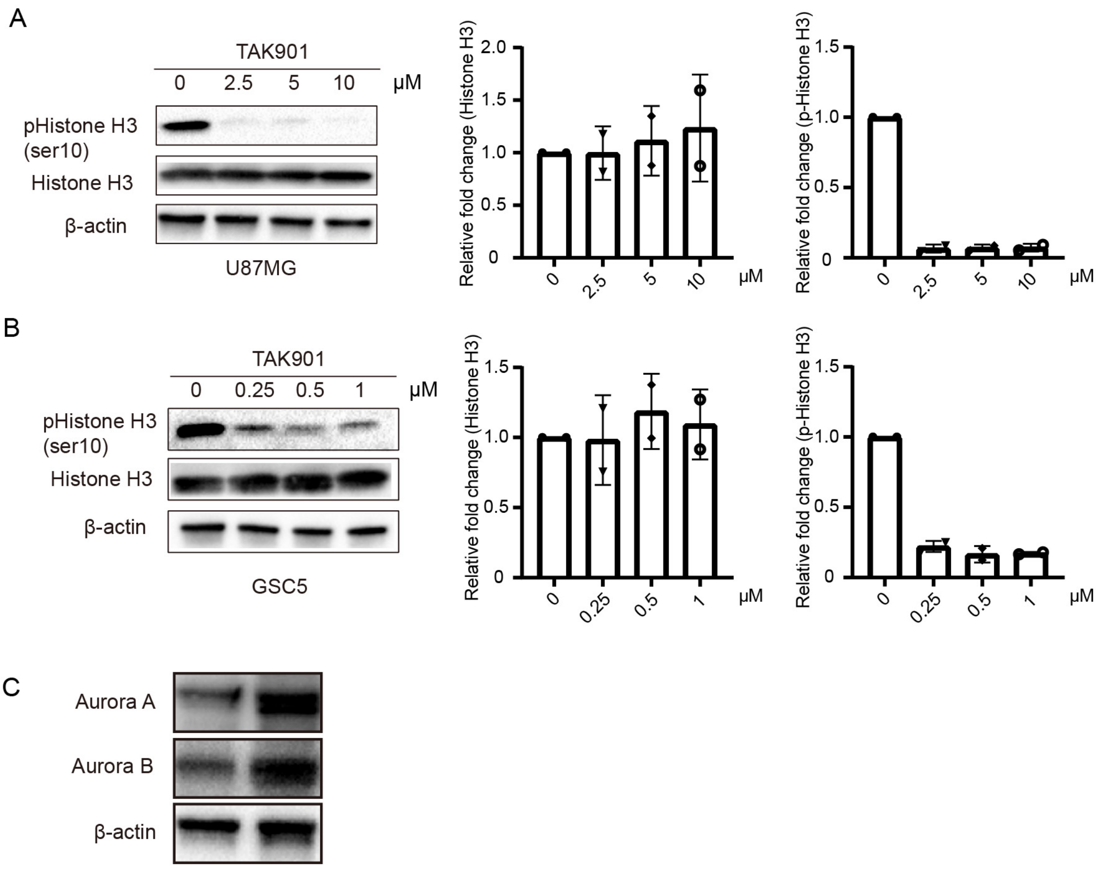
A
TAK901
0 2.5 5 10 μM
pHistone H3 (ser10)
Histone H3
β-actin
U87MG
Relative fold change (Histone H3)
Relative fold change (p-Histone H3)
B
TAK901
0 0.25 0.5 1 μM
pHistone H3 (ser10)
Histone H3
β-actin
GSC5
C
Aurora A
Aurora B
β-actin
